# Nanostructured Pr-Rich Ce_x_Pr_1-x_O_2-δ_ Mixed Oxides for Diesel Soot Combustion: Importance of Oxygen Lability

**DOI:** 10.3390/nano14060483

**Published:** 2024-03-07

**Authors:** Imene Mekki, Gabriela Grzybek, Andrzej Kotarba, Avelina García-García

**Affiliations:** 1 Carbon Materials and Environment Research Group (MCMA), Department of Inorganic Chemistry and Institute of Materials, University of Alicante, Carretera de San Vicente del Raspeig, s/n, 03690 Alicante, Spain; im63@gcloud.ua.es; 2 Faculty of Chemistry, Jagiellonian University in Kraków, Gronostajowa 2, 30-387 Krakow, Poland; g.grzybek@uj.edu.pl (G.G.); kotarba@chemia.uj.edu.pl (A.K.)

**Keywords:** ceria-praseodymia nanoparticles, diesel soot combustion, catalytic activity, oxygen vacancies

## Abstract

Soot combustion experiments with 5%O_2_/He were conducted using model soot, and four distinct compositions of Ce_x_Pr_1-x_O_2-δ_ oxides of varying nominal cerium compositions (x = 0, 0.2, 0.3, and 1) were prepared. The catalyst samples were comprehensively characterized using techniques such as XRD, Raman spectroscopy, HR-TEM, N_2_ adsorption at −196 °C, XPS, O_2_-TPD, H_2_-TPR, and work function measurements. The Pr-rich compositions, ranging from Ce_0.3_Pr_0.7_O_2-δ_ to PrO_2-δ_, resulted in a significant increase in the total evolved O_2_ amounts and enhanced catalyst reducibility. However, a decrease in the textural properties of the catalysts was noted, which was particularly important for the pure praseodymia under the synthesis route conducted. The catalytic activity was investigated under the two following contact modes of mixing between soot and catalyst: *loose* and *tight*. The results revealed that the catalytic performance is associated with the surface contact in tight contact mode and with the combination of surface/subsurface/bulk oxygen mobility and the BET surface area in *loose* contact mode. Notably, the temperatures estimated at 10% and 50% of the conversion (T_10_ and T_50_) parameters were achieved at much lower temperatures than the uncatalyzed soot combustion, even under *loose* contact conditions. Specifically, the 50% conversion was achieved at 511 °C and 538 °C for Ce_0.3_Pr_0.7_O_2_ and Ce_0.2_Pr_0.8_O_2_, respectively. While no direct correlation between catalytic activity and work function was observed, a significant relationship emerges between work function values and the formation of oxygen vacancies, whatever the conditions used for these measurements. On the other hand, the ability to generate a high population of oxygen vacancies at low temperatures, rather than the direct activation of gas-phase O_2_, influences the catalytic performance of Pr-doped ceria catalysts, highlighting the importance of surface/subsurface oxygen vacancy generation, which was the parameter that showed a better correlation with the catalytic activity, whatever the soot conversion value or the mode of contact considered.

## 1. Introduction

In the heavy-duty vehicles and non-road mobile machinery industry, diesel engines are expected to maintain a significant presence alongside alternative powertrain solutions, especially in regions outside the EU. This is driven by the implementation of stringent emission requirements and CO_2_ legislation in Europe beyond 2025, mandating diesel vehicles to achieve optimal fuel economy while emitting zero-impact emissions [1]. The potential Euro 7 emission limit proposed by the Consortium for Ultra-Low Vehicle Emission (CLOVE) aims to develop scenarios for a substantial reduction in real-world emissions while remaining achievable based on the concept of “best-performing vehicles” [2]. Diesel engines are a significant source of particulate matter (PM), which is primarily composed of soluble organic fraction (SOF) and soot. The majority of PM particles are very small, with approximately 90% being less than 1 micrometer and 70% less than 0.3 micrometers [3]. These emissions have adverse environmental and health effects, including the potential to cause cancer [4]. In response to these concerns, many countries have implemented stricter emission regulations. The proposed particulate number (PN) limit by the European Commission (EC) in the latest information on Euro 7 regulation in November 2022 [5] is more stringent than that of Euro VI Step E (9.78 × 10^11^/kWh for PEMS measurement) and is particularly challenging for diesel vehicles. The advancements in the latest generation of diesel particulate filter (DPF) materials have been significant. However, the implementation of stricter particulate regulations necessitates further enhancements in DPF technology. In particular, the proposed PN emission limits for Euro7, covering a broad range of operating conditions and targeting particulate matter as small as 10 nm, as outlined in the CLOVE proposal, underscores the need for continued improvements in particulate number (PN) filtration performance [6].

Wall-flow diesel particulate filters (DPF) with a 90% capture efficiency are commonly used to address PM emissions in diesel vehicles [7]. However, the accumulation of soot inside the DPF can lead to performance deterioration, necessitating regular regeneration to burn out the accumulated soot and reduce back pressure. This is crucial as excessive back-pressure can negatively impact CO_2_ emissions [7], which is a key performance criterion. It is important to note that the self-ignition temperature of soot is higher than the typical exhaust temperature of diesel engines, posing a challenge for effective regeneration. Therefore, the catalyzed Diesel Particulate Filter (cDPF) aids regeneration by reducing the activation energy for soot oxidation. This involves applying an oxidation catalyst to the filter, thus lowering the temperature needed for the combustion of soot. Ceria-based catalysts have been extensively studied as a cost-effective alternative to PGM-based catalysts in this context [3].

The process of catalytic soot combustion involves a unique gas–solid–solid heterogeneous reaction. Consequently, the effectiveness of the catalyst is primarily determined by both its inherent activity and the degree of contact between the catalyst and the soot particles [8]. In this line, Sarli et al. [9] indicated that only low soot loading can make a good contact state with the corresponding catalyst. However, as more soot is loaded into the filter, the contact between the catalyst and the soot weakens [10]. The extent of contact between the two solids is a determinant of the soot combustion activities. Based on this context, recently, a notable number of researchers have been dedicated to improving this degree of contact in the catalysts designed for this application. One of the most studied strategies has been the design of effective and ordered macroporous catalytic structures. It is important to keep in mind that the real soot diameter can be around 25 nm; therefore, constructing ordered macroporous tunnels can be an effective way to enhance the catalyst-soot contact [8], thus further optimizing the catalytic response. Notably, three-dimensional ordered microporous (3DOM) catalysts have been extensively studied for their ability to significantly reduce the transfer resistance of soot particles within their pores. This reduction is attributed to the periodic and orderly arrangement of macroporous structure in 3DOM catalysts, leading to an overall improvement in catalytic activity. However, the lifetime during working conditions and the coating of these structures onto the real DPF remain unclear issues [8].

Another feasible and interesting route to overcome these difficulties is the design of nano-catalysts with an outstanding oxygen delivery capacity, which could partially alleviate the drawbacks of poor contact in a real cDPF. In this sense, ceria has been revealed as a very interesting solid due to its redox behavior, which could be amplified by the insertion of several dopants, among which praseodymium is revealed as one of the most interesting [11]. Praseodymium oxide has an equal structure to that of ceria, with similar crystal parameters. The fact that Pr can easily move among Pr^4+^ and Pr^3+^ oxidation states promotes excellent mobility of oxygen in the bulk and yields excellent soot combustion catalysts, as previously published [11,12]. Nevertheless, the influence of the Pr content on the ceria lattice has not been analyzed in detail at very high Pr contents, where the impact can be much more remarkable. As an example, Andana et al. analyzed nanostructured ceria-praseodymia catalysts until a maximum Ce/Pr composition of 50/50 [11], yielding the best catalytic response, but no additional research was carried out for it, emphasizing the interest in exploring the highest Pr contents.

The need for catalysts with outstanding oxygen delivery capacity and maximum abilities to incorporate oxygen vacancies into their structure is of paramount importance since diesel soot oxidation with oxygen should be promoted mainly by oxygen in present and future diesel vehicles. This is motivated by the last Euro 6/7 standards, which have substantially lowered the allowed NOx emissions. Therefore, and due to the intrinsic features of the whole post-treatment catalytic architecture, it is more efficient to locate the de-NOx system upstream of the cDPF, meaning that, practically, only oxygen remains as a suitable oxidant for the soot combustion process [7].

This study investigates the impact of high oxygen emissions from rich ceria-praseodymia oxides on soot combustion, considering the oxygen delivery capacities and BET surface areas, aiming to unravel the intricate dynamics of how the oxygen released at different temperatures affects soot combustion and how the BET surface area and degree of contact influence the soot combustion process through an in-depth analysis, thus combining the soot combustion profiles versus temperature with the results of soot combustion rates versus soot conversion. For this purpose, a detailed study of the physico-chemical properties of the solids, such as the possibility of the creation of defective ordered structures for the solids synthesized, is explored. Additionally, the work function was employed as a complementary parameter to gain insights into the unique properties of these solids, and the correlation between the work function and the catalytic responses of the catalysts was explored. To facilitate a comparative evaluation of the prepared catalysts, their catalytic activity was benchmarked against a well-established model catalyst, the reference pure ceria.

## 2. Materials and Methods

### 2.1. Catalyst Preparation

Four compositions of Ce_x_Pr_1-x_O_2-δ_ mixed oxides have been prepared with different nominal compositions in cerium and/or praseodymium (x = 0, 0.2, 0.3, and 1; being x referred to as cerium content), including pure praseodymia and ceria, respectively. The solids were prepared by an intimate mixture of Ce(NO_3_)_3_·6H_2_O (supplied by Sigma Aldrich, Fallavier, France, 99%) and/or Pr(NO_3_)_3_·6H_2_O (supplied by Sigma Aldrich, 99.9%) as precursors by taking the required amounts and mixing in a mortar for 10 min. Subsequently, a calcination step at 500 °C for 1 h in static air was carried out. This method was chosen due to the criteria of simplicity and sustainability compared with other more common methods, such as co-precipitation under alkali media [12,13].

### 2.2. Catalyst Characterization

BET surface areas were measured by multi-point N_2_ adsorption at −196 °C using an automatic Autosorb-6B (Quantachrome equipment, Quantachrome Instruments’ corporate headquarters, Boynton Beach, FL, USA). The samples were previously degassed for 4 h at 250 °C under vacuum.

X-ray diffractograms of the samples were recorded in a Bruker D8 advanced diffractometer using CuKα radiation with a wavelength of 0.15406 nm. Spectra were registered between 10° and 80° (2 h) with a step of 0.1° and a time per step of 3 s. Indexation of the patterns was accomplished using dedicated software (DIFFRAC.EVA, https://www.bruker.com/es/products-and-solutions/diffractometers-and-x-ray-microscopes/x-ray-diffractometers/diffrac-suite-software/diffrac-eva.html, accessed on 17 March 2022), and the average crystal sizes were calculated using Scherrer’s equation.

The Raman spectra were obtained using a LabRam Jobin Ivon Horiba Raman Spectrometer equipped with a confocal microscope and a variable-power He-Ne laser source emitting light at 633 nm with a laser power of 1 mW. Each spectrum was acquired by performing 2 scans, each lasting 200 s.

XPS, a K-Alpha spectrophotometer (Thermo-Scientific), with a high-resolution monochromator spectrometer, was employed with an Al anode (1253.6 eV) radiation source. The binding and kinetic energy scales were adjusted by considering the C transition signal at 284.6 eV. The surface composition of the catalysts was analyzed using the Ce-3d and Pr-3d regions, along with the C-1s and O-1s regions. To determine the proportion of Ce^3+^ cations compared to the total cerium present on the surface, the calculation method outlined by Laachir et al. [14]. was employed. Similarly, the relative percentage of Pr^3+^ cations to the total praseodymium was estimated following the approach suggested by Borchet et al. [15].

The sample morphology was analyzed at a microscopic level using transmission electron microscopy methods. The equipment used was the FEI Tecnai Osiris, equipped with an X-FEG Schottky field emitter (200 kV) and a high-angle annular dark-field (HAADF) detector (Karlsruhe Institute of Technology, Karlsruhe, Germany). Elemental mapping was conducted using Energy Dispersive X-ray (EDX) spectroscopy, employing a Super-X EDX windowless system with a 4-sector silicon drift detector (SDD). The distribution of the elements in the catalysts under investigation was obtained using the GMS 3.43.3213.0 

The O_2_-temperature-programmed desorption under He (O_2_-TPD) was performed with a simultaneous TG-DTA coupled with a mass spectrometer (TG-DTA-MS). A 20 mg sample of the catalyst was pretreated under helium flow (100 mL/min) at 150 °C for 1 h, then heated under programmed temperature from 150 °C to 950 °C at 10 °C/min.

Temperature-programmed reduction with H_2_ (H_2_-TPR) was performed using a Micromeritics Auto Chem II 2920 apparatus. In each trial, 20 mg of catalyst was placed in a U-shaped quartz tube and pretreated with a flow of 5% O_2_/He, and the samples were heated up to 500 °C (10 °C/min) for 1 h. After cooling down the samples, the gas was changed to a 10% H_2_/He flow (40 mL/min). The signal from a thermal conductivity detector was recorded from 30 to 950 °C at a linear heating rate of 10 °C/min.

The work function values (Φ) of the investigated catalysts were evaluated based on measurements of the contact potential difference (V_CPD_) carried out by the Kelvin method with a KP6500 probe (McAllister Technical Services), as described in detail elsewhere [16]. The reference electrode was a typical stainless-steel plate with a diameter of 3 mm (Φref ≈ 4.3 eV). The work function values were obtained from a simple relation: eV_CPD_ = Φref − Φsample. To ensure data reproducibility, the work function values were calculated as an average of 3 independent measurements for each sample.

### 2.3. Catalytic Soot Combustion Tests

The soot combustion reaction was evaluated by using a thermogravimetric analyzer (Mettler Toledo TGA/DSC) with an Al_2_O_3_ crucible. Non-isothermal TG tests were carried out by using a heating ramp of 10 °C/min up to 900 °C under 5% O_2_/He. The catalyst/soot ratio employed was 20/1 under different contact modes; the so-called *tight contact* (T.C.) conditions were obtained by mixing the powders well in a mortar for 10 min. The reasons for this choice were a higher level of reproducibility and more meaningful catalytic activity data [17]. On the other hand, the second procedure consists of mixing the components with a spatula (in a very gentle way for 4 min) to afford the *loose contact* mode (L.C.), which is the most realistic one. The catalytic activity data were estimated from three independent measurements, and the average errors about characteristic temperatures given in this work can be estimated as ±3 °C.

## 3. Results and Discussion

### 3.1. Catalyst Characterization

#### 3.1.1. Structural and Textural Parameters

**Figure 1:**
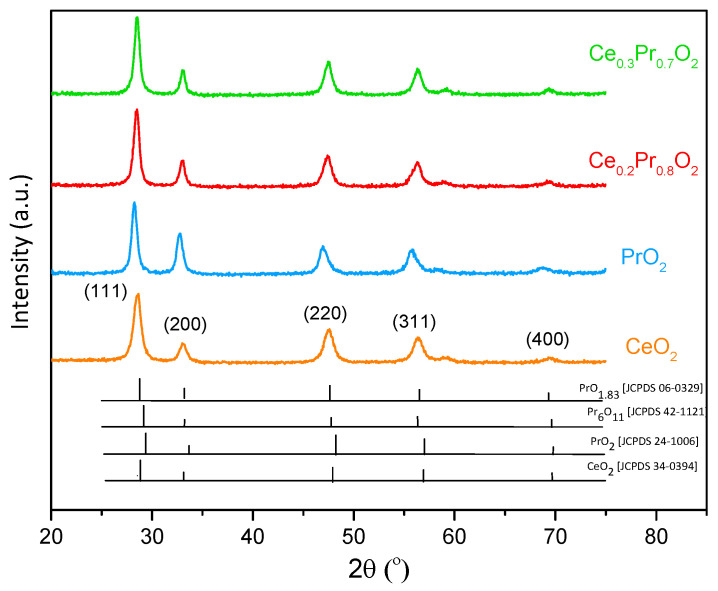
X-ray diffractograms of the investigated catalysts, together with the corresponding references.

Figure 1 shows the X-ray diffractograms of the four catalysts prepared. The pure ceria exhibits a single cubic fluorite structure 12, as expected, with four typical reflections corresponding to the (111), (200), (220), and (311) planes, observed at 28.55, 33.05, 47.50, and 56.40°, respectively [12,13]. The three Pr-based formulations seem to exhibit a single cubic fluorite structure as well [13]. Nevertheless, the diffractograms of Pr-containing samples are very complicated to interpret because Pr can form several stoichiometric and non-stoichiometric sub-oxides with the formula PrOx, where x ≤ 2, which would present diffraction patterns only slightly different from those of fluorite ceria. However, the shift of PrO2 peaks toward lower angles with regard to the position of pure ceria peaks was fully consistent with the formation of PrO1.83 or Pr6O11 phases (JCPDS files: 06-0329 and 42-1121). The two corresponding mixed oxides are found in an intermediate situation.

Additionally, the lattice parameter (*a*) can be obtained for each catalyst by using Bragg’s law (and determining the d parameter), while Scherrer’s equation is employed for the average crystal size estimation. All these results are summarized in Table 1. The values slightly increase by considering a progressive substitution of cerium by praseodymium into the cationic sublattice, providing evidence of a higher presence of the Pr^3+^ cation until reaching the pure praseodymium oxide formulation limit [12]. On the other hand, quite similar average crystal sizes can be observed for the four formulations. Conversely, a lowering in the BET surface area is seen inside the series and is quite noticeable for pure praseodymia (6 m^2^/g). A previous study [13] reported a negative effect of very high Pr loadings on the ceria (concerning BET surface area and pore development) when the direct calcination method was employed. In fact, recent works by Frizon et al. [18] and Fahed et al. [19] reported that the insertion of high Pr loadings in mixed oxides (specifically CZ-mixed oxides) significantly decreased the specific surface area. The observation of the non-correlation between S_BET_ and average crystal size (in spherical approximation; r(nm) = 3/(ρ·S_BET_)), where ρ is the compound density, was attributed to the fact that the particles were not spherical and the aggregation phenomena occurred, limiting the pore size (see Table 1). High Pr loading can decrease the inter-granular porosity in line with the drop in porous volume observed from the data in Table 1.

**Figure 2:**
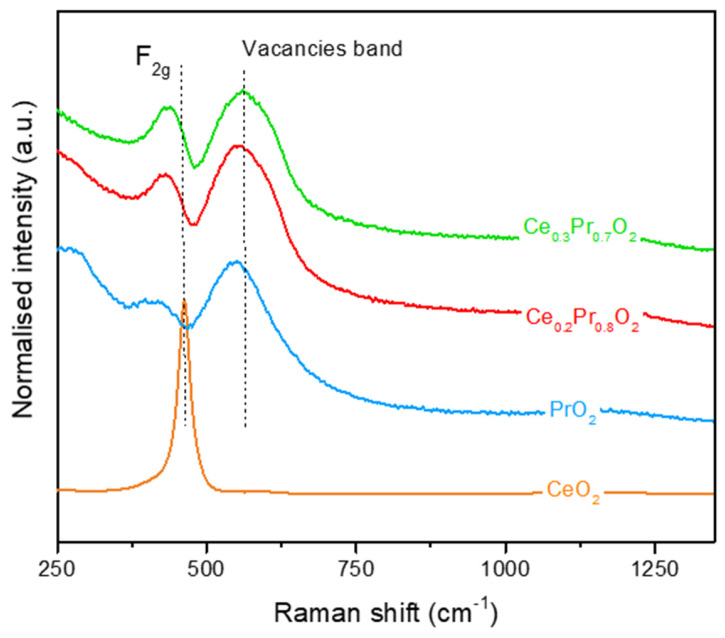
Raman spectra of the investigated Pr-containing catalysts.

Raman spectroscopy is a highly suitable technique for the structural characterization of cerium-based oxides, providing additional structural information beyond that offered by the XRD technique. The XRD patterns provide information mostly about the positions of large cations in the crystal lattice (such as cerium). However, this technique offers very low sensitivity to light elements (such as oxygen) [12]. Raman spectra of ceria revealed the *F_2g_* band at 464 cm^−1^, which might be attributed to a symmetrical breathing mode of the oxygen ions combined with the tetravalent cations [20,21]. The three Pr-containing catalysts show the F_2g_ vibration mode of the fluorite structure (see Figure 2), where the band position is moved to lower wavenumber values by the addition of Pr content onto ceria, as listed in Table 1, where this decrease can be due to the existence of the trivalent cation (rather Pr^3+^ than Ce^3+^), i.e., a weaker force constant of the corresponding cation-anion bonds. This interpretation is also supported by the observed mode at a wavenumber of about 570 cm^−1^, which is attributed to a defect mode caused by oxygen vacancies, as mentioned in a previous publication [12]. The relatively high intensity of this mode under atmospheric conditions indicates the presence of the reduced cations (rather being Pr^3+^ than Ce^3+^). The vacancy band/F_2g_ band ratio (I_560_/I_F2g_) obtained from the normalized spectra correlates with the Pr content, as can be seen in Table 1. This ratio can be considered an indicator (or descriptor) of the abundance of oxygen vacancies caused by the substitution of Ce atoms by Pr atoms in the mixed oxides until achieving pure praseodymium oxide [20].

**Figure 3:**
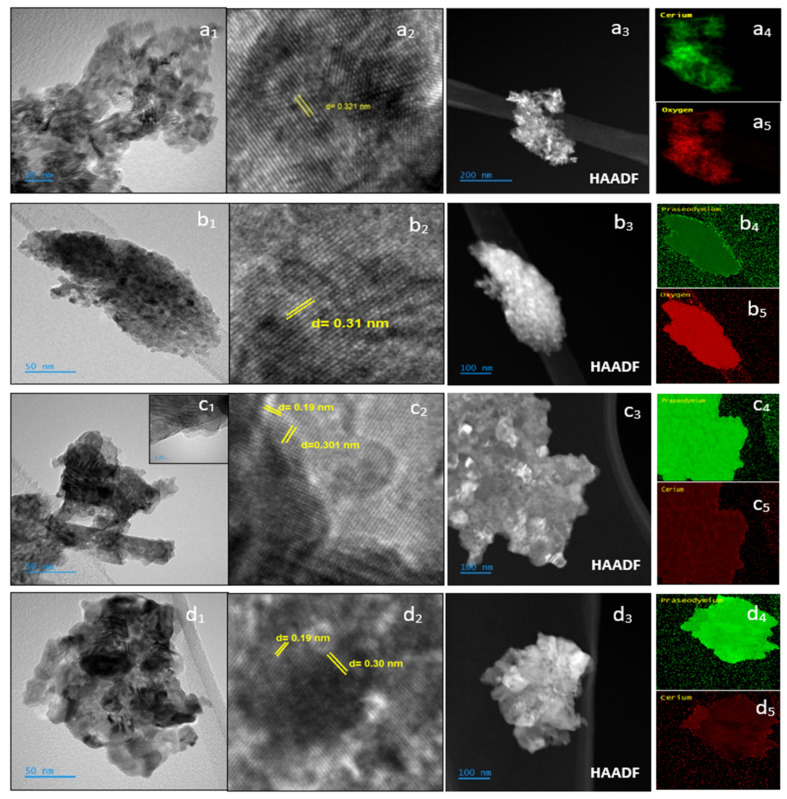
Representative TEM (**a_1_**,**b_1_**,**c_1_**,**d_1_**), HR-TEM (**a_2_**,**b_2_**,**c_2_**,**d_2_**), and HAADF-EDX (**a_3_**–**d_3_**,**a_4_**–**d_5_**) analysis for the investigated catalysts: (**a_1_**–**a_5_**) for CeO_2_, (**b_1_**–**b_5_**) for PrO_2_, (**c_1_**–**c_5_**) for Ce_0.2_Pr_0.8_O_2_, and (**d_1_**–**d_5_**) for Ce_0.3_Pr_0.7_O_2_.

In order to explore if the catalysts present varied morphologies dependent on the molar composition of cerium and praseodymium, the high-resolution TEM images of the catalysts investigated are illustrated in Figure 3. The presence of aggregates of multiple nanoparticles can be generally observed: a dense substance made of nanocrystalline domains that have been sintered exhibits a significant number of likely disordered grain boundaries. Although the individual particles are not visible in Figure 3(a_1_–d_1_) (this might be due to the route of synthesis used, as reported in a previous study [13]), there is strong evidence of crystallinity within the domains, as seen by the visible lattice fringes (Figure 3(b_2_–d_2_)) and even clearer in Figure 3(a_1_–a_4_), corresponding to the pure ceria catalyst (analyzed for comparison purposes). The interplanar distances of the lattice fringes observed are compatible with the fluorite structure of the catalysts, showing (111) fringes (close to 0.31 nm) [22,23] and (220) facets with an interplanar distance of 0.19 nm as shown in Figure 3(b_2_,c_2_) [22,23], which confirms the XRD results, where the reflections corresponding to both planes are the most intense ones detected. Actually, the d value obtained for the (111) facets, measured for the three catalysts, exhibits a slight and progressive increase in the corresponding value (0.30, 0.301, and 0.31 nm, respectively) as the Pr loading enhances from Ce_0.3_Pr_0.7_O_2_ to PrO_2_, in complete agreement with the *a* (lattice parameter) values measured by XRD, where this value is also increased. It should be noted that the crystal structure of ceria and praseodymia shows large similarities [23]. Therefore, phase segregation is difficult to infer (if any), and according to these results, there is no evidence of such phenomena [15].

The representative HAADF images in Figure 3(a_3_–d_3_) of CeO_2_, PrO_2,_ Ce_0.2_Pr_0.8_O_2,_ and Ce_0.3_Pr_0.7_O_2_, respectively, show the appearance of spherical agglomerates in the mixed oxide. These images corresponded well with the energy dispersive spectroscopy (EDS) element mapping images of Ce (a_4_–c_5_ and d_5_), Pr (b_4_–c_4_ and d_4_), and O (a_5_–b_5_) showing relatively high-density distributions, where there is a homogenous distribution of cerium and praseodymium on the nanometric scale over the Ce_0.2_Pr_0.8_O_2_ and Ce_0.3_Pr_0.7_O_2_ catalysts, which is also consistent with the TEM and XRD results.

Surface-sensitive X-ray photoelectron spectroscopy (XPS) has been used to study the surface chemical state of mixed oxide particles. Table 2 shows some representative parameters obtained from the XPS spectra analysis (see Figures S1 and S2 in the Supplementary Materials).

The obtained results suggest that the Ce/Pr surface atomic ratios were consistently lower than theoretical or nominal values in ceria-praseodymia samples. This indicates that there was a higher concentration of praseodymium on the particle surface (or the periphery of the particles) than that of cerium (thus exhibiting Pr surface enrichment) [12]. When conducting estimations, the Ce 3d and Pr 3d levels were utilized, both of which do not exhibit significantly different binding energies. The Ce 3d level has a binding energy of approximately 870 eV, while the Pr 3d level has a binding energy of around 970 eV. These values indicate the amount of energy required to remove an electron from the respective 3D orbitals of cerium (Ce) and praseodymium (Pr) atoms [24]. Overall, the segregation of dopant cations at the surface and domain boundaries of ceria is an important aspect to consider when studying or utilizing ceria-based materials, as it can have significant implications on their properties and performance in various applications [19,25]

Pr cations (Pr^3+^ and Pr^4+^) are incorporated into the cerium oxide lattice (and/or partially segregated at the particle’s surface), as suggested by the comparison of the Ce/Pr surface values with the nominal ones. Therefore, the presence of nano-domains with different Ce/Pr compositions (indistinguishable by XRD and/or HR-TEM) cannot be excluded for the two mixed oxides. Corresponding estimations are compiled in Table 3.

The expected and well-described “carbonation” process that occurs on solid surfaces during calcination and storage of samples can pose challenges for the analysis and interpretation of Ce^3+^ and Pr^3+^ cations’ predominance [12,13]. This process leads to the accumulation of carbon on the surface of these oxides, as indicated in Table 2. The presence of carbon species on the surface can make the accurate determination of the Ce^3+^ and Pr^3+^ cations (truly involved in the creation of oxygen vacancies) quite difficult. The estimated values of Pr^3+^ (%) are always higher than those of Ce^3+^ (%) for the two mixed oxides prepared, which are the expected results since Pr is a more reducible cation (due to the higher reduction potential and more labile Pr-O bond) and is more basic in nature compared to Ce [23,24,26]. Additional details concerning XPS analysis can be found in SI.

#### 3.1.2. Comparison of the Oxygen Lability of the Catalysts under Different Environments

The redox properties and the corresponding lability of the oxygen species in these formulations were explored by means of two procedures that can complement each other: H_2_-TPR and O_2_-TPD (to measure the O_2_ evolved under an inert atmosphere). For more details about definitions, procedures, and estimations conducted to determine the amount of oxygen vacancies created after the two different procedures, please see the Supplementary Materials.

**Figure 4:**
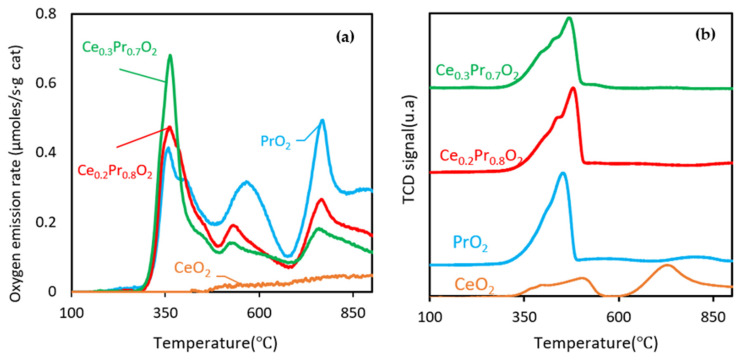
(**a**) O_2_-TPD and (**b**) H_2_-TPR profiles of the catalysts.

O_2_-TPD under an inert atmosphere (helium) was used to examine the oxygen “delivery” of the catalysts and, in turn, their lability under helium by following the rate of oxygen desorption in terms of temperature (and its subsequent global quantification, thus obtaining the value of µmol O_2emitted_/g_cat_). Figure 4a displays the O_2_-TPD profiles of the catalysts, expressed as the mentioned oxygen emission rate versus temperature, and Table 4 compiles the corresponding quantifications. Even though CeO_2_ is a well-known oxygen storage material, O_2_ desorption under an inert atmosphere was hardly observed in this case, in line with previous results [13], as shown in Figure 4a. Conversely, the three other samples (the Pr-containing catalysts) yielded three main and large oxygen desorption peaks from 300 °C to 900 °C, whose general pattern is very similar (differing mainly in the relative contribution of every peak), which seems to be a function of the Pr content. Since the two mixed oxides’ general profile is very similar to that obtained for the pure oxide, the discussion about the oxygen mobility can be assessed in a more certain way due to the presumed absence of separate rich Pr and/or Ce domains in the solids, which could probably modify the oxygen emission pattern, thus making a distinction if referred to that of the pure oxide. It is generally expected that the existence of such large domains would display different oxygen labilities. This indirect evidence would be congruent with the XRD and TEM discussion, where a main crystalline phase with a gradual variation of the lattice parameter was observed when going from the Ce_0.3_Pr_0.7_O_2_ sample to PrO_2_.

The sharpest peak and the highest values of O_2_ emission rates in the low-temperature range are reached by Ce_0.3_Pr_0.7_O_2_ at 363 °C (this temperature maximum is very similar for the three catalysts), which can be attributed, in principle, to the most surface/reactive oxygen susceptible to being evolved. The profile and magnitude of this first peak, being the sharpest and most intense one of the whole pattern for the two mixed oxides, reveal an accentuated oxygen lability and, in turn, relevant oxygen mobility at very low temperatures, which can be assumed to be very influenced by the BET surfaces since the magnitude and maximum oxygen emission rate at this very low temperature follow the same trend as that of the BET surface area and pore volume of the solids. Conversely, the second and third peaks are more accentuated for pure PrO_2_, revealing the maximum bulk oxygen mobility and the best data on global oxygen evolved for this formulation. Therefore, the higher the Pr loading, the higher the O_2_ evolved amounts, which is in line with the conclusions of other authors [11,13].

For the deepest insight, some quantitative considerations will be provided now. Considering a mean oxygen density of 21.7 µmol O·m^−2^ in ceria-based oxides, according to the assumptions taken by Fahed et al. [19] based on other authors [27], the oxygen emitted relative to the first peak (see values in parentheses of Table 4) only concerns surface oxygen species (or a fraction of them) for the two mixed oxides under study. Conversely, for the case of PrO_2_, more than the theoretical oxygen surface population (even though it is small due to its very low surface area) can be desorbed in this low range of temperature, proving evidence of the very high oxygen mobility at the surface/subsurface level for this catalyst.

It is interesting to approach the whole oxygen delivery capacity deduced from these experiments (O_2_-TPD). If it is bear in mind that the ideal reduction of PrO_2_ to Pr_2_O_3_ would yield a maximum amount of oxygen evolved of 1445 μmol O_2_/g_cat_ (indicating the maximum oxygen released that could be reached by a bulk oxide of this starting formulation, fully oxidized) [28], the value of 1045 μmol O_2_/g_cat_ for pure praseodymia (even characterized by a very low surface area) indicates very high lability of oxygen and an accentuated mobility of subsurface/bulk oxygen even under a not reducing atmosphere (He).

H_2_-TPR was used to assess the reducibility of the selected catalysts as well as determine the amount of oxygen vacancies susceptible to being created in a different reaction atmosphere. Figure 4b depicts the profiles of H_2_ consumption obtained by this procedure. It is interesting to compare, as deeply as possible, both Figure 4a,b. Firstly, the profiles under H_2_ are characterized by similar patterns (in agreement with the previous discussion) and asymmetrical shapes, the higher the Pr content in the catalysts, the sharper and more intense the peak. This has a certain parallelism with the O_2_-TPD profiles, where the three different peaks can be interpreted as the “delivery” of more and more internal lattice oxygen, but they are now appearing as a unique peak (but with weak shoulders, under a more aggressive atmosphere, H_2_) [12]. From a very basic and qualitative perspective, it seems that the three previous peaks, detected under He, have been combined in a single asymmetric peak that decays at an early temperature (around 500 °C). The corresponding integration of this peak (in Figure 4b) for every catalyst confirms the increasing reducibility (ability to form oxygen vacancies) as the Pr loading is progressively enhanced in the catalysts’ formulation [12].

The delta (δ) parameter has been used to indicate that a single oxygen-deficient non-stoichiometric phase is formed. In this sense, the general formula after the corresponding reduction will be written as MO_2-δ_ or Ce_1-x_Pr_x_O_2-δ_ to express the maximum level of non-stoichiometry that has been reached under the two different atmospheres (He or H_2_). For example, for PrO_2_, its maximum theoretical level of non-stoichiometry would be δ equal to 0.5 [28,29], which corresponds to the reduced formula of PrO_2-0.5_ or PrO_1.5_ (better written as Pr_2_O_3_), where the whole Pr^+4^ cations have been reduced to Pr^+3^).

The values of δ, or non-stoichiometry, reached, together with the final formula after the reduction of the different catalysts under the two atmospheres, are shown in Table 4. An increasing trend of δ is observed with the increase in the content of praseodymium, reflecting the better reducibility and, in turn, improved oxygen mobility in both atmospheres since the oxygen amount evolved is very high (and not only affecting the surface oxygen, as previously commented and explained below in more detail). Furthermore, as the Pr content is increased, the capacity of oxygen delivery under the two atmospheres becomes closer and closer, thus reflecting superior oxygen lability as well, which is suggested to be of paramount importance for the soot combustion application.

**Figure 5:**
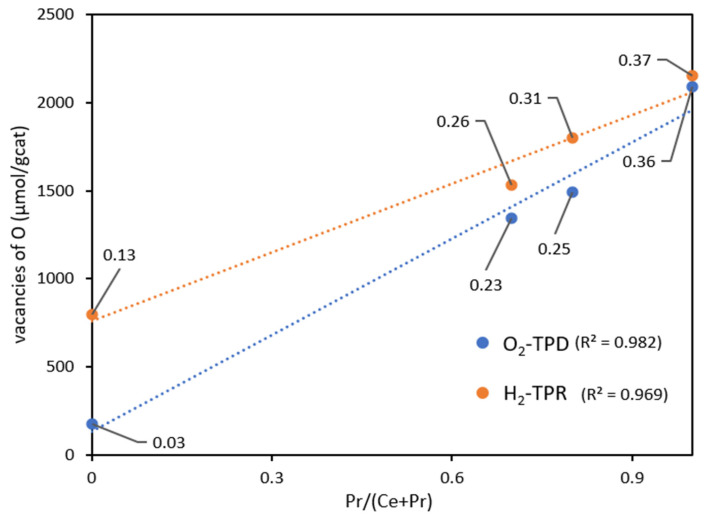
Oxygen delivery capacity, expressed as the amount of oxygen vacancies formed under different atmospheres, versus Pr loading (along with estimated δ values for every catalyst).

Figure 5 illustrates the correlations of the amounts of oxygen vacancies created under the two atmospheres (estimated from global O_2_ emitted and H_2_ consumed amounts shown in Table 4 and following the assumptions explained in the Supplementary Materials) versus the praseodymium content of the catalysts (ranging from pure ceria to pure praseodymia). It is worth noting that, independently of the different textural properties that characterize the catalysts, the main parameter governing the total oxygen release capacity of the oxides is the praseodymium content (approximately linear correlation, see Figure 5), mainly that obtained by the representation of O_2_-TPD data, with a value of r^2^ of 0.982. Furthermore, the following interesting observation can be extracted from Figure 5: as the Pr content is higher and higher, the values obtained under the two different atmospheres become closer and closer, proving the best oxygen mobility and, in turn, the high ability of stabilizing vacancies for PrO_2_ (even under mild conditions).

Interestingly, for the case of pure praseodymia, the maximum theoretical level of reducibility that can be reached (measured from both atmospheres) corresponds with the value of the theoretical non-stoichiometric phase of Bevan’s cluster. It is described in the literature [29] that fluorite-related solids (MO_2-x_) can be anion deficient, with the cation sublattice remaining essentially perfect. The defect cluster would correspond to tightly bound vacancies along a body diagonal (111) (as shown in Figure S3 of the Supplementary Materials). The central cation is six-coordinated and is surrounded by six seven-coordinated cations. Since the geometry of these clusters permits four different equivalent orientations, it is easily accommodated in the fluorite structure and accounts for the wide range of stability of the so-called α-phases (MO_1.71_-MO_2_). This congruency between the experimental estimation and the theoretical defect cluster mentioned proves the large population of bulk oxygen vacancies accommodated in this solid, thus leading to a bulk defect cluster structure and becoming indirect evidence of the large capacity of stabilizing oxygen vacancies into the lattice after mild or reducing atmospheres.

### 3.2. Catalytic Performances of Soot Combustion under 5% of the O_2_/He Atmosphere

O_2_-TPD experiments have proved a large ability to release O_2_ at very low temperatures for the Pr-containing catalysts and, consequently, are available for catalytic reactions when compared with the very poor response coming from the ceria catalyst. To assess the impact of these behaviors on the catalytic soot combustion, due to the different BET surface areas and pore volumes characterizing the catalysts, Temperature-Programmed-Oxidation (TPO) experiments under 5%O_2_/He, both in tight and loose contact conditions, were carried out.

The accepted mechanism of soot oxidation over ceria-based solids involves the formation of oxygen vacancies at the interface between the catalyst and the soot. These vacancies can act as sites for oxygen activation, forming reactive species that will spill over the soot surface to oxidize the corresponding C atoms. This mechanism clearly depends on the degree of contact between the catalyst and the soot, because this impacts the population of oxygen vacancies that will form at the interface, where soot can play a role as a reductant for the ceria-based oxide, and the spilling of active O species, which is facilitated by a large contact surface area. The number of contact points in the interfacial region is of paramount importance to promote soot oxidation at low temperatures; these will be highly dependent on the textural parameters of the catalysts, mainly in the first stages of the reaction [30]. In this sense, the soot combustion experiments were conducted under the two typical configurations (*tight* and *loose* contact) [30]. The data obtained from both configurations could provide deeper knowledge about the performance of the catalysts and the possibility of “splitting” and determining the impact of BET surface area, surface oxygen mobility, and bulk oxygen mobility on catalytic performance.

#### 3.2.1. Experiments in Tight Contact Mode

It is widely accepted that *tight contact* conditions are less representative of the real situation concerning the contact achieved among soot and catalyst in a real DPF [31], but the advantages of this mode of mixture are as follows: good reproducibility, reliable results, and a preliminary view or “screening” of the intrinsic catalytic behavior. It is definitely very helpful to reflect the inherent features of a catalyst. Nevertheless, the order of activities should be carefully discussed since the trends (when different catalysts are compared) and the performances found could be very different from those obtained under the *loose-contact* mode.

**Figure 6:**
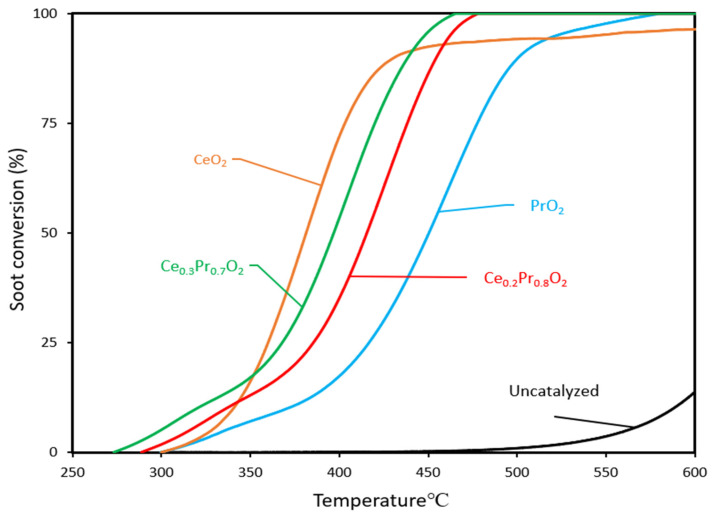
Soot conversion curves (under *tight contact*) as a function of temperature for all the catalysts (including the uncatalyzed reaction, for comparison purposes).

Firstly, the catalytic activity of the investigated samples is represented by the soot conversion curves as a function of temperature (and corresponding T_10%_, T_20%_, T_50%,_ and T_90%_ values, which are listed in Table 5). To provide a basis for comparison, the profile of pure cerium oxide (taken as a model catalyst) is included as a reference, and all the profiles are shown in Figure 6. It is interesting to pay attention to the region of low temperatures (or low soot conversion values), where the initial catalytic beds are in similar starting conditions since the soot loaded has not been consumed in remarkable amounts. The catalytic activity can be ranked in the following sequence:

Ce_0.3_Pr_0.7_O_2_ > Ce_0.2_Pr_0.8_O_2_ > PrO_2_ ~ CeO_2_

Ceria exhibits the highest BET surface area measured (81 m^2^/g) of the series, followed by Ce_0.3_Pr_0.7_O_2_, Ce_0.2_Pr_0.8_O_2_, and PrO_2_, respectively. However, even at the initial steps of the reaction, the BET surface area seems not to be the most important parameter governing the activity. Paying attention to Figure 4a, one can see that this is the order of oxygen lability at low temperatures for Pr-containing samples (the order of BET surface areas and the activities match as well); meanwhile, pure ceria presents a very poor ability to release oxygen under O_2_-TPD conditions (nearly null).

As soon as the reaction proceeds and the temperature is increased, CeO_2_ is characterized by higher soot conversion values comparing the same range of temperature (from 350 °C on) with the Pr-containing samples, which present very good oxygen mobility [12,32]. This observation is consistent with the results published by Aneggi et al. [32], who reported superior activity for pure ceria than that of Zr-doped ceria (with good bulk oxygen mobility), analyzing soot combustion activities in air under tight contact conditions. This indicated, according to the authors, that the regeneration of surface oxygen by sub-surface or bulk oxygen was not prevailing and the replenishment of surface oxygen was mainly due to the O_2_-gas phase, because no correlation was found with the respective values of oxygen storage capacity (OSC) of the catalysts [32]. The present results seem to point in a similar direction.

**Table 5:** Comparison of the temperatures needed to achieve several values of conversion (X%) along with the soot combustion rates (in parentheses) measured under tight and loose contact conditions (T.C. and L.C.).

Sample	Temperature (°C)							
	10% ^a^		20% ^a^		50% ^a^		90% ^a^	
	T.C.	L.C.	T.C.	L.C.	T.C.	L.C.	T.C.	L.C.
CeO_2_	340 (0.89) ^b^	439 (0.24) ^b^	355 (1.46) ^b^	483 (0.43) ^b^	382 (2.23) ^b^	556 (0.83) ^b^	415 (0.80) ^b^	630 (0.51) ^b^
Ce_0.3_Pr_0.7_O_2_	318 (0.36) ^b^	342 (0.30) ^b^	357 (0.71) ^b^	409 (0.32) ^b^	397 (1.78) ^b^	511 (0.78) ^b^	438 (1.07) ^b^	595 (0.69) ^b^
Ce_0.2_Pr_0.8_O_2_	334 (0.38) ^b^	359 (0.28) ^b^	373 (0.63) ^b^	431 (0.27) ^b^	415 (1.74) ^b^	538 (0.94) ^b^	454 (1.20) ^b^	618 (0.58) ^b^
PrO_2_	367 (0.24) ^b^	404 (0.31) ^b^	405 (0.72) ^b^	469 (0.15) ^b^	449 (1.54) ^b^	555 (0.72) ^b^	500 (0.63) ^b^	632 (0.58) ^b^
1Ag/YSZ ^c^	380	420	-	-	430	530	-	-

^a^ % soot conversion values. ^b^ soot combustion rates (expressed as mg_soot_·s^−1^·g_initial soot_^−1^ in parentheses). ^c^ extracted data from [33] (formulation with 6 m^2^/g of BET surface area).

**Figure 7:**
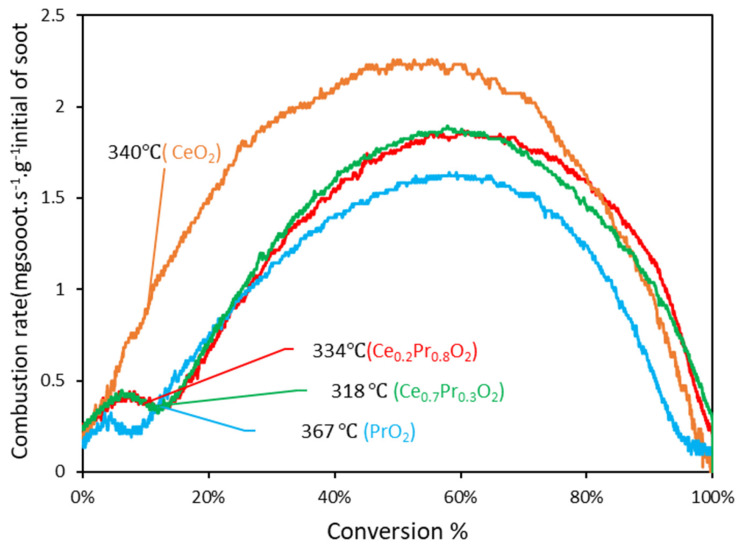
Evolution of soot combustion rates versus soot conversion values along the experiments conducted under *tight contact* mode (the values of temperature for every catalyst taken for 10% of conversion).

In order to work with more accurate data and reach better comparability, the evolution of soot combustion rates was depicted versus the soot conversion values in Figure 7. With these representations, the catalytic activity data are much more comparable since the analysis is based on the same amount of remaining soot in the reactor. Interestingly, soot combustion rates seem to be higher at low conversions for the ceria catalyst, which could be considered contradictory results. Nevertheless, this is only a consequence of the delay in appreciable soot combustion rates at low temperatures (probably due to the absence of a good oxygen delivery capacity of this catalyst). When going deeper into the information in Figure 7 and the corresponding temperatures at which the soot conversions reached are taken into account (see values compiled in Table 5), it can be clearly checked that the highest soot combustion rate measured at iso-conversion of 10% for the pure ceria catalyst is due to the highest temperature needed compared with the Pr-containing mixed oxides (340° versus 318/334 °C) and not to a better intrinsic activity. In this line, the comparative representations of Figure 7 (along with the summary compiled in Table 5) are quite useful and needed for a reliable discussion of the results. From 20% of conversion on, ceria is the catalyst exhibiting the highest soot combustion rate at the lowest temperature, which is likely attributed to its highest BET surface area, which provides more surface-active sites for the combustion reaction to take place at medium temperatures. However, pure ceria’s profile decays before, indicating that this catalyst is seen as much more affected than Pr-containing mixed oxides by the loss of contact between soot particles and the catalyst’s surface, and, from 80% of conversion on, the ceria’s rate drops below that of the Pr-containing mixed oxides.

#### 3.2.2. Experiments in Loose Contact Mode

The experiments conducted under *tight contact* conditions, discussed previously, do not reflect the real conditions achieved in a real coated diesel particle filter, where the contact between soot and catalyst is often very poor, and this affects the efficiency of the catalytic process (which can be considered optimum in the *tight contact* mode).

**Figure 8:**
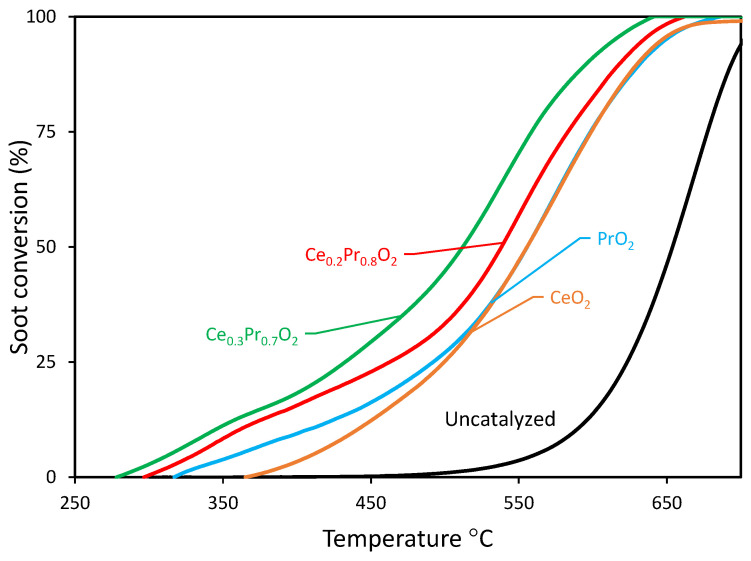
Soot conversion curves (under loose contact) as a function of temperature for all the catalysts (including uncatalyzed reactions, for comparison purposes).

Figure 8 shows the soot conversion curves in *loose contact* mode as a function of temperature for each catalyst. As expected, these combustion profiles are shifted towards lower temperatures with regard to the uncatalyzed profile. On the other hand, the soot combustion profiles are clearly moved towards higher temperatures if compared with those obtained under *tight contact* mode and are characterized by an activity order considerably different as follows:

Ce_0.3_Pr_0.7_O_2_ > Ce_0.2_Pr_0.8_O_2_ > PrO_2_ > CeO_2_

(if attention is paid, mainly, to the range of low and medium temperatures).

According to this ranking, Ce_0.3_Pr_0.7_O_2_ exhibited the highest catalytic activity among the catalysts tested, followed by Ce_0.2_Pr_0.8_O_2_. The use of Ce_0.3_Pr_0.7_O_2_ resulted in the highest soot conversion in the initial temperature range, while CeO_2_ showed the lowest conversion rate. In general, ceria’s performance is seen to be much higher than that of Pr-containing samples if compared with the trends under *tight contact* mode, emphasizing the idea that pure ceria would be much less active under real conditions. A tentative explanation can be given now: as soon as the catalyst-soot contact becomes poorer and poorer (*loose contact* versus *tight contact* mode), the role of a large population of oxygen that can be released in the proper range of temperature becomes more relevant. As seen by the O_2_-TPD profiles, ceria presents a very low population of oxygen species that can be released under He and, therefore, is able to contribute to the soot combustion process. Conversely, the Pr-containing samples are much more active than ceria (mainly in the low-temperature range), just following the order of oxygen emission degree in the low-temperature range (see Figure 4a).

**Figure 9:**
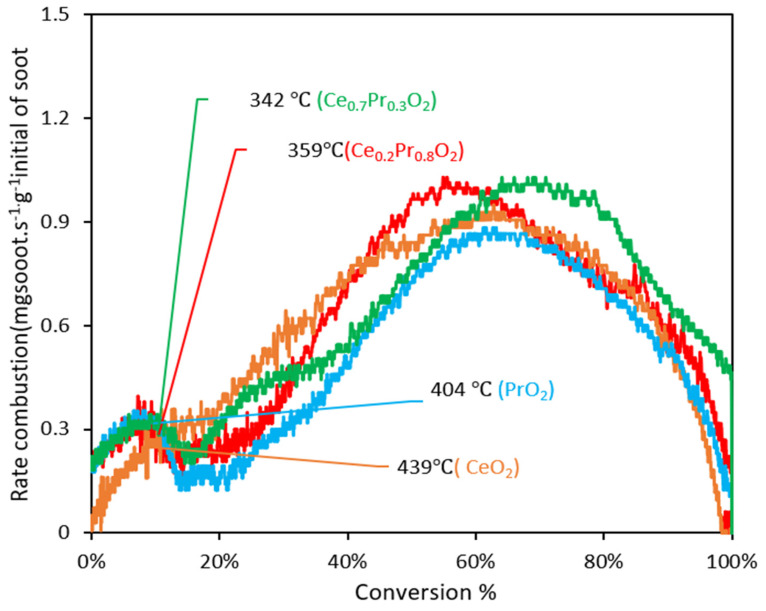
Evolution of soot combustion rates versus soot conversion values along the experiments conducted under *loose contact* mode (the values of temperature for every catalyst are taken for 10% of conversion).

The evolution of soot combustion rates was illustrated versus the soot combustion values in Figure 9, in parallel with the analysis made in *tight contact* mode. From a comparative point of view, it is worth commenting on the general profiles of this set of catalysts under the two situations tested. Much higher maximum values and higher differences in the catalysts’ behaviors are shown by means of conducting experiments under *tight contact* (if compared with those of *loose contact*), thus verifying that, effectively, the *tight contact* conditions can be defined as a mode of mixture able to reveal the intrinsic differences in the catalytic performances. The very intimate degree of contact maximizes the soot combustion rate, and, probably, the relevant steps of the soot combustion process can play a “harmonized” role (since there are no constraints between the catalyst surface and the soot surface joints); therefore, as soon as the oxygen species are replenished by O_2_-gas, they are effectively transferred to the soot’s surface.

A detailed analysis of the data in Figure 9 reveals that the pure ceria sample achieves the same soot conversion rates at identical values of conversion but at different temperatures compared to the Pr-containing samples. This trend aligns with the order of the oxygen emission ability at low temperatures as determined by O_2_-TPD. The data collected in Table 5 confirms that the ceria catalyst always needs a higher temperature to achieve the same soot conversion values, compared with whatever Pr-containing catalyst is considered (under more or less comparable soot conversion rates). Only pure praseodymia, which presents a considerably low BET surface area and pore volume, is characterized by similar values of temperature to those of ceria at high conversion values. All these findings are supported by the fact that the Pr-containing catalysts present a tremendous ability to deliver high oxygen amounts at low temperatures, and a part of them will eventually reach the soot surface, even though direct contact among the surfaces is hindered.

The discussion of this work is completed by the direct comparison of the soot conversion profiles for every catalyst (in separate graphs). Therefore, in order to obtain a more comparative and reliable analysis, the curves obtained under the two modes (*tight* and *loose*) are compared for each catalyst in Figure S4 of the SI, in an attempt to analyze which catalyst (or catalysts) are seen to be more affected by the decrease in the number, quality, and intensity of the contact points (catalyst/soot), or, in other words, the change from an intimate contact mode to a gentle or more representative contact mode. By means of these representations, the following general statement can be assessed: better performances were observed at the highest degree of contact, whatever the nature of the catalyst; however, remarkable differences can be noted in the patterns, and the relative differences in activity in terms of the contact mode are a function of the nature of the catalyst.

Therefore, Figure S4 illustrates the impact of the contact mode (*tight* vs. *loose*) on the soot conversion profiles and highlights important observations previously mentioned. The behavior of ceria in both contact modes, *tight* and *loose*, differs significantly when compared to that of the praseodymium-containing formulations. These formulations exhibit two distinct slopes, indicating a possible change in the rate-determining step (or mechanism). Conversely, the ceria catalyst does not show two distinct slopes, but a conventional sigmoidal shape appears. This change of slope is clearly reflected in the relative maximum appearing at low conversion values, which features the representations of Figure 7 and Figure 9 for Pr-containing catalysts but is completely absent for the pure ceria catalyst.

One potential explanation for this difference lies in the different performance or features of the ceria’s catalyst. In this case, the catalytic activity is thought to primarily depend on the surface oxygen species’ population, created in the course of the reaction. In contrast, for the praseodymium-containing formulations, at certain temperatures, the catalytic activity appears to depend on the surface/subsurface/bulk oxygen species’ mobility (besides the involvement of surface oxygen).

This observation suggests that the praseodymium-containing formulations have improved oxygen mobility (as proved by O_2_-TPD and H_2_-TPR results), enabling efficient active oxygen transfer from the catalyst to the soot surface. This improved oxygen transfer mechanism is likely due to the enhanced mobility of surface/subsurface/bulk oxygen species in these formulations. This ability seems to be especially critical in loose contact mode.

On the other hand, the differences between tight and loose contact curves are the lowest in the case of PrO_2_. Actually, this catalyst presents the lowest BET surface area (6 m^2^/g) and the best bulk oxygen mobility. As the Pr-containing catalysts are characterized by higher and higher surface areas, the corresponding curves move further away from each other, mainly in the low-temperature range (as shown by Ce_0.2_Pr_0.8_O_2_ (31 m^2^/g) and Ce_0.3_Pr_0.7_O_2_ (42 m^2^/g), respectively).

Finally, to assess the interest of these compositions against other catalysts’ formulations recently reported in the literature [33], Table 5 compiles the measured values of T_10%_ and T_50%_ obtained from soot combustion curves under quite similar conditions (tight and loose contact modes; 5%O_2_/He; 100 mL/min; and Printex-U as model soot) by choosing the most active formulation reported by Serve et al. (1Ag/YSZ), extracted from [33]. The impact of the present results is notorious since the two mixed oxides are more active than the selected catalyst, 1Ag/YSZ, for whatever the conditions are compared. Interestingly, this catalyst (presenting a BET area of 6 m^2^/g and a V_p_ of 0.02 cm^3^/g) shows quite similar textural features to those of pure praseodymia (see Table 1), and even the pure praseodymia catalyst is more active than the Ag-catalyst at low conversion values, which can be again supported by the large ability to deliver oxygen at low temperatures of this catalyst. Accordingly, the benefits of Pr-containing samples, among other formulations, seem to be more evident at low temperatures.

### 3.3. Work Function Measurements

A crucial initial stage in many catalytic reactions involves the transfer of an electron from the catalyst surface to the reacting molecules. The material’s capacity to perform this electron transfer is determined by its work function, which is essentially the energy required to extract an electron from the material’s surface. This electron transfer can generate active entities that are more inclined to engage in subsequent reactions or modify the charge of a molecule, enabling it to interact with a molecule of opposite charge [16]. According to the literature [4,17,34], the work function of the catalyst can be manipulated either by substituting elements in its core structure or by introducing surface promoters. It is well known that promoting the use of alkali materials with transition metal oxides leads to a reduction in the work function of the parent materials, resulting in an active catalyst.

Electronic surface promotion could play an important role in the catalytic oxidation of soot, as it is related to the activation of gas-phase oxygen, responsible for the combustion of carbonaceous pollutants. However, a direct relationship between catalytic activity (either under loose or tight contact) and the work function values could not be found under the experimental conditions used in this study. This suggests that the formation of reactive oxygen species (such as O^−^, O_2_^−^, and O_2_^2−^) on the catalyst surfaces via electrons may not be of key importance for the investigated catalysts.

**Figure 10:**
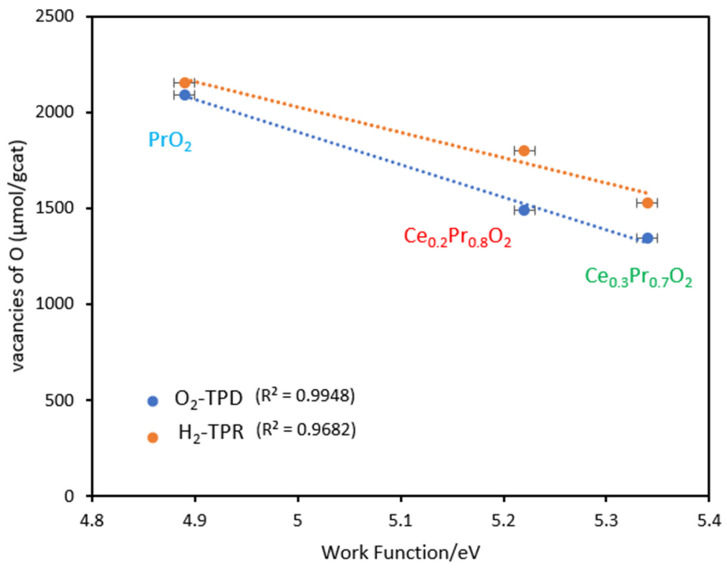
Relationship between the total oxygen vacancies generated (measured by O_2_-TPD and by H_2_-TPR) and the work function values for the Pr-containing catalysts studied. (Error bars added for the corresponding work function measurements).

Indeed, an intriguing aspect emerges when a possible relationship between the work function values and the formation of oxygen vacancies under the two tested atmospheres is examined. There appears to be a noticeable correlation between the amounts of oxygen vacancies created and the Pr-rich catalyst’s work function, as illustrated in Figure 10. Specifically, the sample with the highest ability to generate oxygen vacancies, considering the whole range of temperatures (pure praseodymia), is that with the lowest value of work function. The regression coefficient obtained is very close to 1 (0.9948) for the data obtained from O_2_-TPD (see Figure 10).

**Figure 11:**
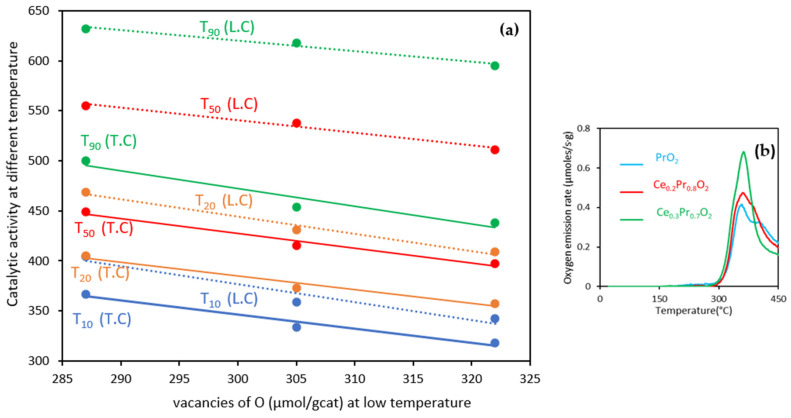
Catalytic activity data, expressed as temperatures needed to achieve several conversion degrees under both loose (L.C.) and tight contact (T.C.), versus the number of vacancies determined at low temperatures from O_2_-TPD (by using the profiles represented in (**b**)).

Conversely, the catalytic activity might be tentatively related to the oxygen emission capacity in the low-temperature range (Figure 11a). Since the soot amounts remaining in the reactor are not the same at the different temperatures of the study, the temperatures to achieve different degrees of conversion (see Table 5) were chosen to be represented against the estimation of the potential oxygen vacancies created at low temperatures (see Figure 4a and specific representation on Figure 11b). All the lines present reasonable correlations, but the best regression coefficients are obtained mainly for loose contact conditions, indicating that the activity is influenced by the contact mode, and at loose contact mode, the impact of the oxygen emitted at low temperatures (corresponding to the first peak of O_2_ emission) is more determinant in defining the orders of catalytic activities.

As a brief summary, the comparison of soot conversion profiles under TPR mode should be carried out in a very careful and detailed way since the soot combustion rates must be compared at the same or similar soot conversion values and even temperatures, mainly in the new scenarios of legislation where the exhaust temperatures will become lower and lower. The TPR profiles of the catalysts conducted under the two contact modes provide valuable information about their activity and performance in diesel soot oxidation, and comparing these profiles under consistent conditions is essential for meaningful comparisons. The Pr-rich compositions seem to be promising formulations for real diesel soot combustion applications due to their very high oxygen lability, if compared with traditional pure ceria, characterized by poor oxygen lability at medium temperatures.

## 4. Conclusions

Based on the obtained results, the following conclusions can be drawn:

Mixed Ce-Pr oxides of cubic fluorite structure prepared via direct calcination exhibit a large population of oxygen vacancies. The associated high non-stoichiometry values estimated from TPD-O_2_ under an inert atmosphere (which is slightly increased under a H_2_ atmosphere, depending on the catalyst formulation) are strongly dependent on the Pr content (as the Pr content increases, the reducibility increases). Specifically, an increase in Pr content correlates with enhanced oxide reducibility. Conversely, pure ceria presents a very poor oxygen delivery capacity.

The order of catalytic activity shown by the oxides is very sensitive to the mode of contact. A high oxygen delivery capacity at low temperatures is a determinant to explain the activity under *loose* contact mode. Conversely, BET surface area seems to play a role in explaining the soot combustion activity, especially at medium temperatures in tight contact mode.

In a loose contact situation (in addition to the replenishment of gaseous O_2_ for regeneration of surface-active oxygen), subsurface and/or bulk diffusion of oxygen from the lattice sites also becomes an important pathway, resulting in increased activity of Pr-doped ceria under O_2_ in comparison to undoped CeO_2_. A temperature of 653 °C was needed to achieve 50% soot oxidation in the uncatalyzed process, whereas the presence of Ce_0.3_Pr_0.7_O_2_ and Ce_0.2_Pr_0.8_O_2_ dramatically decreased the T50% to much lower temperatures, even under *loose* contact conditions. Specifically, the 50% conversion was achieved at 511 °C and 538 °C for Ce_0.3_Pr_0.7_O_2_ and Ce_0.2_Pr_0.8_O_2_, respectively, revealing the satisfactory performances of these formulations under loose contact mode.

Although the catalytic activity for the Ce-Pr oxide catalysts does not correlate directly with the work function, a relationship has been observed between the work function values and the formation of oxygen vacancies under the two tested atmospheres (He and H_2_). This strongly suggests that for praseodymium-based catalysts, the formation of reactive oxygen species on the surface via electron transfer is not of primary importance, and soot combustion is dominated by the Mars-van Krevelen mechanistic pathway.

Some guidelines for future research are as follows:

The relevant results obtained in this study regarding Pr-rich Ce_x_Pr_1-x_O_2-δ_ mixed oxides demonstrate their very high reactivity towards soot combustion without NOx and their excellent oxygen delivery capacities at low temperatures. These findings can be applied to future work on gasoline direct injection (GDI) soot combustion, where soot needs to be oxidized in the absence of appreciable oxygen, as it is only available during fuel cuts in GDI exhaust. These demanding exhaust conditions may present opportunities for the utilization of these Pr-based materials.

## Figures and Tables

**Table 1:** Selected textural and structural parameters of the catalysts.

Sample	Lattice Parameter *a* (nm)	Average Crystal Size (nm)	S_BET_ (m^2^/g)	Pore Volume (V_p_) (cm^3^/g)	F_2g_ Band Position (cm^−1^)	Vacancies Band/F_2g_ Band (Intensity Ratio)
CeO_2_	0.5415	11.1	81	0.221	463.1	-
Ce_0.3_Pr_0.7_O_2_	0.5418	12.2	42	0.059	438.6	1.09
Ce_0.2_Pr_0.8_O_2_	0.542	12	31	0.03	431.8	1.15
PrO_2_	0.5467	11.5	6	0.012	428.3	1.2

**Table 2:** Surface atomic compositions determined from XPS results.

Sample	Ce (%)	Pr (%)	O (%)	C * (%)
CeO_2_	25.03	-	55.45	19.5
Ce_0.3_Pr_0.7_O_2_	4.94	14.77	50.02	30.25
Ce_0.2_Pr_0.8_O_2_	3.52	14.98	47.38	34.1
PrO_2_	-	13.66	43.01	43.31

* High carbon contents due to residual contamination inherent to the measurements, but mostly to the surface “carbonation” of the oxides.

**Table 3:** Surface atomic ratios and oxidation state quantifications from XPS.

Sample	Pr^+3^ (%)	Ce^+3^ (%)	Ce/Pr_surface_	Ce/Pr_nominal_	O/(Ce + Pr)
CeO_2_	0	36.3	-	-	2.21
Ce_0.3_Pr_0.7_O_2_	48.3	31.1	0.26	0.43	2.53
Ce_0.2_Pr_0.8_O_2_	44.9	34.1	0.23	0.25	2.56
PrO_2_	44.7	0	-	-	2.67

**Table 4:** Oxygen released quantifications (obtained from O_2_-TPD) and H_2_ consumption quantifications (obtained from H_2_-TPR) for the catalysts studied.

Sample	O_2_ Emitted (μmol/g_cat_)	δ_1_	^1^ Formula after Reduction	H_2_ Consumption (µmol/g_cat_)	δ_2_	^2^ Formula after Reduction
CeO_2_	88	0.03	CeO_1.97_	779	0.13	CeO_1.87_
Ce_0.3_Pr_0.7_O_2_	671/(322) *	0.23	Ce_0.3_Pr_0.7_O_1.77_	1530	0.26	Ce_0.3_Pr_0.7_O_1.74_
Ce_0.2_Pr_0.8_O_2_	745/(305) *	0.25	Ce_0.2_Pr_0.8_O_1.75_	1797	0.31	Ce_0.2_Pr_0.8_O_1.69_
PrO_2_	1045/(287) *	0.36	PrO_1.64_	2155.6	0.37	PrO_1.63_

* Oxygen emitted with the quantification in parentheses (only the first peak). ^1^ Formula obtained after reduction in He atmosphere. ^2^ Formula obtained after reduction in H_2_ atmosphere.

## Data Availability

Data are contained within the article and Supplementary Materials.

## Supplementary Materials

The following supporting information can be downloaded at: https://www.mdpi.com/article/10.3390/nano14060483/s1. Figure S1: XPS spectra of: (a) Pr 3d and (b) Ce3d levels; Figure S2: XPS spectra of O 1s level; Figure S3: Bevan cluster in MO_2-x_ oxides of fluorite structure; Figure S4: Soot conversion profiles versus temperature under the two contact modes. (Tight contact: solid lines, and loose contact: dashed lines): (a) CeO_2_; (b) Ce_0.3_Pr_0.7_O_2_; (c) Ce_0.2_Pr_0.8_O_2_; and (d) PrO_2_.; Figure S5: Comparative between fresh and spent catalysts (Ce_0.3_Pr_0.7_O_2_): (a) H_2_-TPR profiles and (b) O_2_-TPD profiles. References [15,29,35,36,37,38,39,40] are cited in the Supplementary Materials.
