# Supplementary material for: Nanostructured Pr-Rich CexPr1-xO2-δ Mixed Oxides for Diesel Soot Combustion: Importance of Oxygen Lability
Source: Nanomaterials (Basel). 2024 Mar 7;14(6):483. doi: 10.3390/nano14060483 (PMC10975723; doi:10.3390/nano14060483)
Supplement: Supplementary file 1 [file nanomaterials-14-00483-s001.zip › nanomaterials-2895196-supplementary.pdf]

Supplementary Information

Nanostructured Pr-rich $Ce_xPr_{1-x}O_{2-\delta}$ mixed oxides for diesel soot combustion. Importance of oxygen lability.

Imene Mekki [1], Gabriela Grzybek [2], Andrzej Kotarba[2] and Avelina García-García [1,*]

[1] Carbon Materials and Environment Research Group (MCMA), Department of Inorganic Chemistry and Institute of Materials, University of Alicante, Carretera de San Vicente del Raspeig, s/n, 03690, San Vicente del Raspeig, Alicante, Spain.
[2] Faculty of Chemistry, Jagiellonian University in Kraków, Gronostajowa 2, 30-387 Krakow, Poland
* Correspondence: a.garcia@ua.es; Tel.: (+34 965909419; A.G.G.)

1. XPS analysis

Figure S1.a shows the Pr3d profiles of the catalysts studied, the Pr 3d spectrum contains 7 peaks organized in 3 spin-orbit coupling doublets, named a-b, a'-b' and a''-b'' and one Auger peak at high binding energy, belonging to the 3d3/2 sublevel. The spectrum Pr3d for each catalyst indicates that Pr^{3+} and Pr^{4+} coexisted. Figure. S2.b exhibits the XPS spectra of Ce 3d of the catalysts. Ce 3d XPS spectra are well known to be complicated due to the hybridization of the Ce 4f orbitals with the ligand orbitals and the fractional occupation of the valence 4f orbitals and the O 2p states [1,2]. These spectra were decomposed into ten contributions. The peaks were labeled according to the standard nomenclature, in agreement with the literature[[3]. Peaks associated with the Ce $3d_{5/2}$ state are labeled v, whereas those associated with the Ce $3d_{3/2}$ state are labeled u. However, the contributions v, v'', v''', u, u'' and u''' belong to Ce^{4+} 3d states indicating the main valence state of Ce in the sample as +4; while the v_0, v', u_0 and u' are attributed to the valence state of Ce^{3+} [3,4].

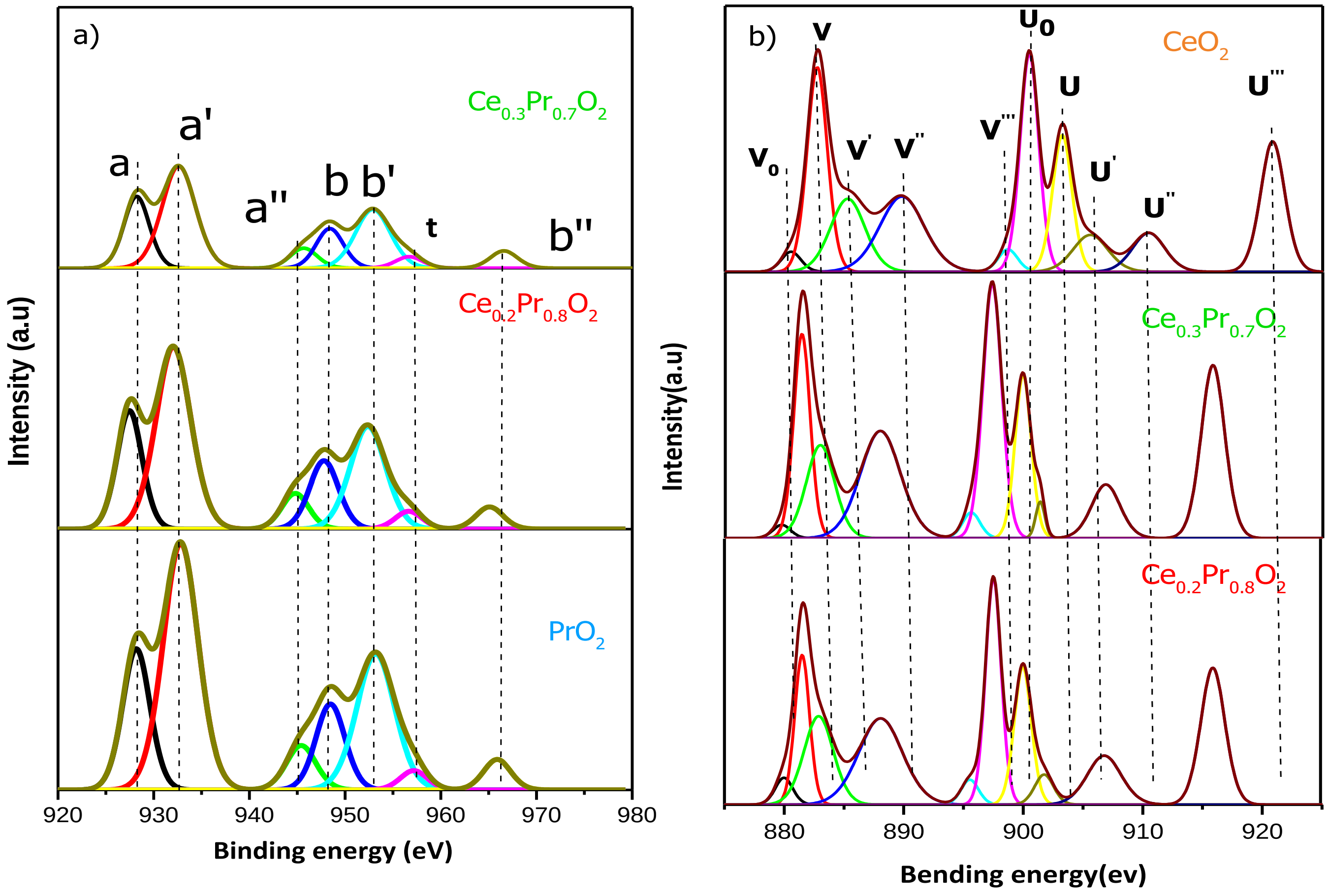

Figure S1. XPS spectra of: (**a**) Pr 3d and (**b**) Ce3d levels

A detailed analysis of the surface oxygen was presented in Figure S2, where the O 1s energy range was depicted for the catalysts. All O 1s profiles showed two peaks, which were attributed to lattice oxygen (low energy peak) and to "loosely-bound" oxygen (carbonates, hydroxyls, adsorbed oxygen, peroxide and superoxide groups… (high energy peak). A large contribution of O_{ads} versus O_{latt} is compatible with a high population of carbonate/oxycarbonates species supported by the high atomic C contents and the O/Ce+Pr ratios higher than 2 for these samples. This assignation based on previous works [5] is more appropriate than other assignations following different criteria but referred to other types of oxides [6].

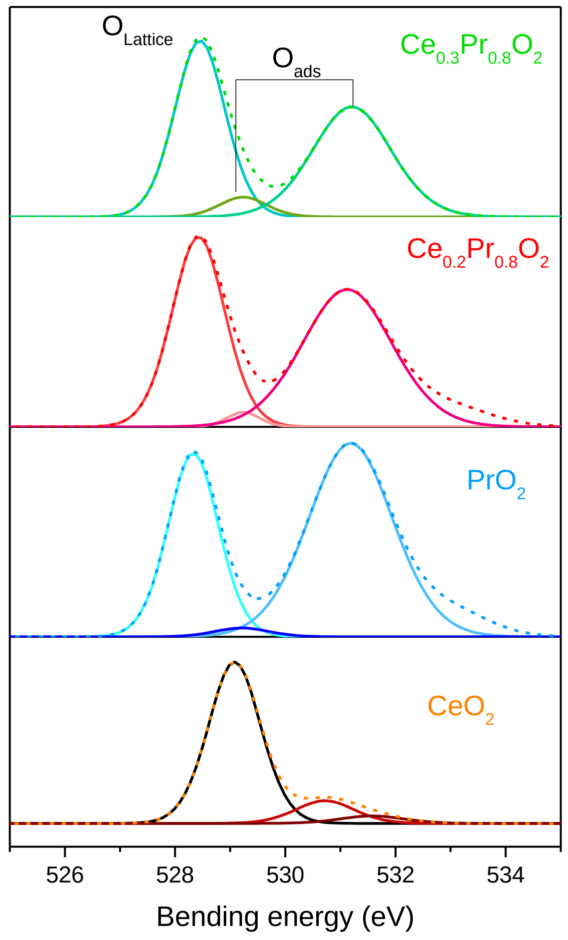

Figure S2. XPS spectra of O 1s level

2. Details on the procedure and estimation of the number of oxygen vacancies.

The number of oxygen vacancies created in the surface/subsurface/bulk of the solids under the different treatments was estimated by assuming that the amount of oxygen released after the different treatments (either as H_2O from H_2-TPR or as O_2 from O_2-TPD under He) proceeds from the surface/lattice oxygen corresponding (eventually) to the creation of the oxygen vacancies by assuming the following global stoichiometries (Reaction 1 and 3).

Therefore, the H_2 consumption (µmol/g_{cat}) estimation is the same as the H_2O emitted during the experiment and agrees with the µmoles O/g_{cat}, corresponding to the oxygen vacancies generated during the experiment (according to the stoichiometry of reaction 1).

By means of H_2-TPR:

$$H_2 + 2MO_2 \rightarrow H_2O + M_2O_3 \qquad \text{R.1.}$$

(by using the calibration of CuO as a standard and assuming that one mole of H_2 is consumed for each mole of CuO to reduce the total Cu^{2+} to Cu^0 according to the following reaction):

$$CuO + H_2 \rightarrow Cu + H_2O\ ) \qquad R.2$$

By means of O_2-TPD:

$$MO_2 \rightarrow MO_{2-\delta} + 1/2\delta O_2 \qquad R.3$$

the O_2 emission (µmol/g_{cat}) would be double the amount of the estimation of µmoles O/g_{cat}, corresponding to the oxygen vacancies generated during the experiment (according to the stoichiometry of Reaction 3).

(by using the calibration of CuO, where the stoichiometric reduction/decomposition of CuO to Cu_2O emitting O_2 occurs under an inert atmosphere, according to the reaction 4)

$$2\ CuO \rightarrow Cu_2O + 1/2O_2) \qquad R.4$$

Oxygen delivery capacity is, the capacity of the catalysts to emit oxygen, and in turn, to create oxygen vacancies in their structures.

3. Theoretical non-stoichiometric phase of the Bevan's cluster

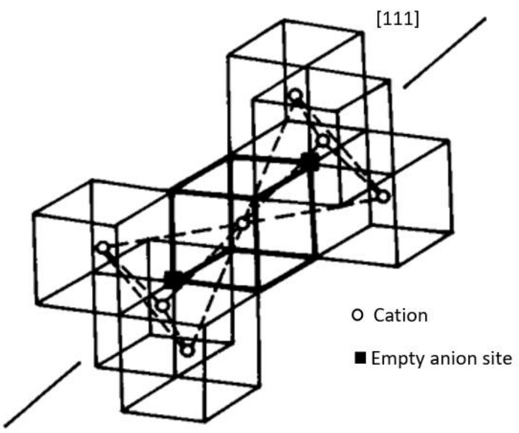

Figure S3. Bevan cluster in MO_{2-x} oxides of fluorite structure. Adapted from reference [7].

4. **Direct comparison of the soot conversion profiles for every catalyst (in separate graphs).**

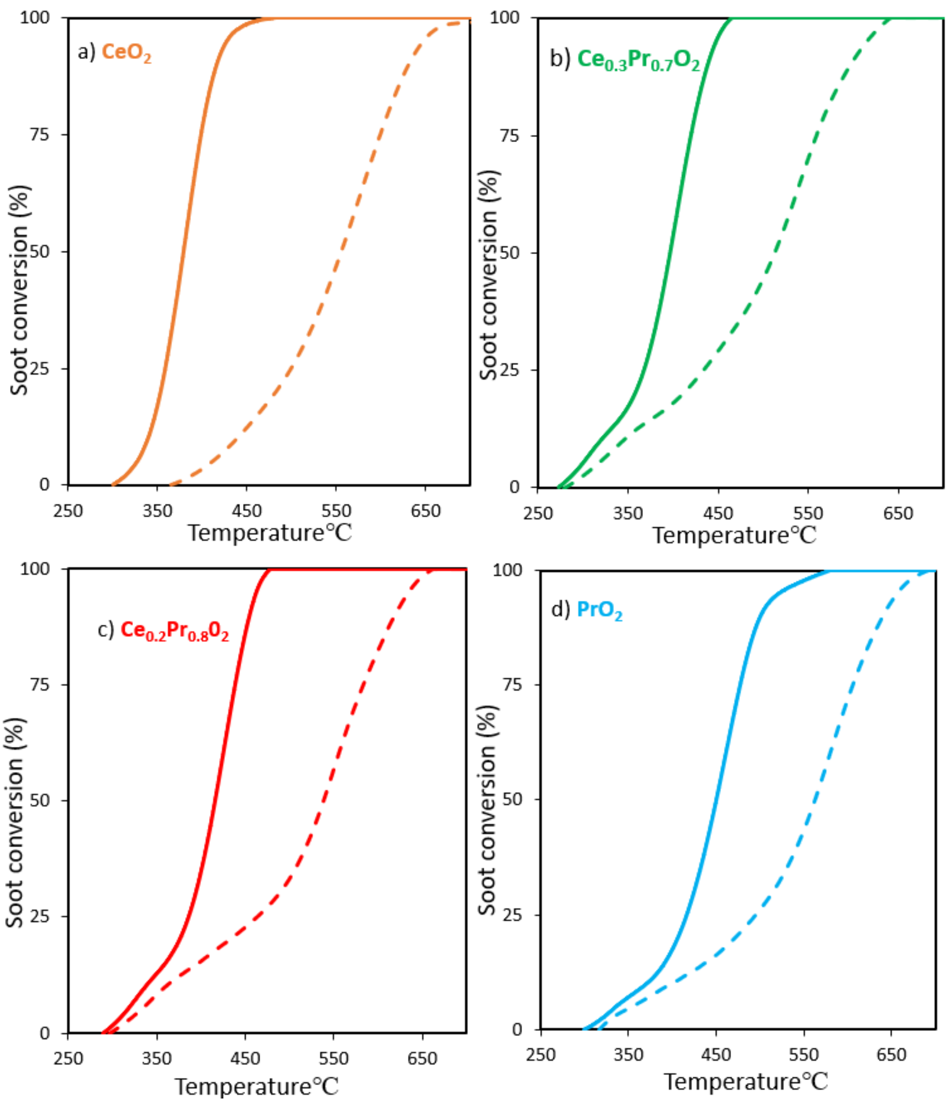

Figure S4. Soot conversion profile*s* versus temperature under the two contact modes. (*Tight contact*: solid lines and *loose contact*: dashed lines): (**a**) CeO_2; (**b**) $Ce_{0.3}Pr_{0.7}O_2$; (**c**) $Ce_{0.2}Pr_{0.8}O_2$ and (**d**) PrO_2.

5. **Additional data on activity, reusability and features of fresh/spent catalysts.**

The catalysts studied are very stable regarding reusability. For instance, when examining pure ceria under specific conditions, a slight decrease in activity (around 5%) was observed from the first to the second cycle of a catalytic tests. This decrease was attributed to a minor loss of BET surface area caused by the high calcination temperature (500°C) during sample preparation, with subsequent testing reaching temperatures up to 750°C. Notably, from the second cycle onwards up to the third cycle, the catalytic performance remained constant, indicating excellent reusability and stability of these formulations [8].

Concerning physico-chemical features of fresh versus spent catalysts and beginning with the comparison of H_2-TPR results of fresh and used $Ce_{0.3}Pr_{0.7}O_2$ (the most active catalyst), the results show that a slight loss in reducibility is suffered by the spent catalyst according with a shift towards higher temperatures evidenced by the H_2-TPR profiles. This is compatible with a very moderate and expected loss in BET surface area, because the calcination temperature involved in sample preparation is 500°C and the corresponding catalytic activity test is extended up to 750°C. Nevertheless, the quantification of H_2 consumption, or in other words, the estimation of oxygen vacancies created under H_2 is nearly the same (see corresponding values in Figure S5.a), proving additional evidence of the interest of these formulations for the desired application.

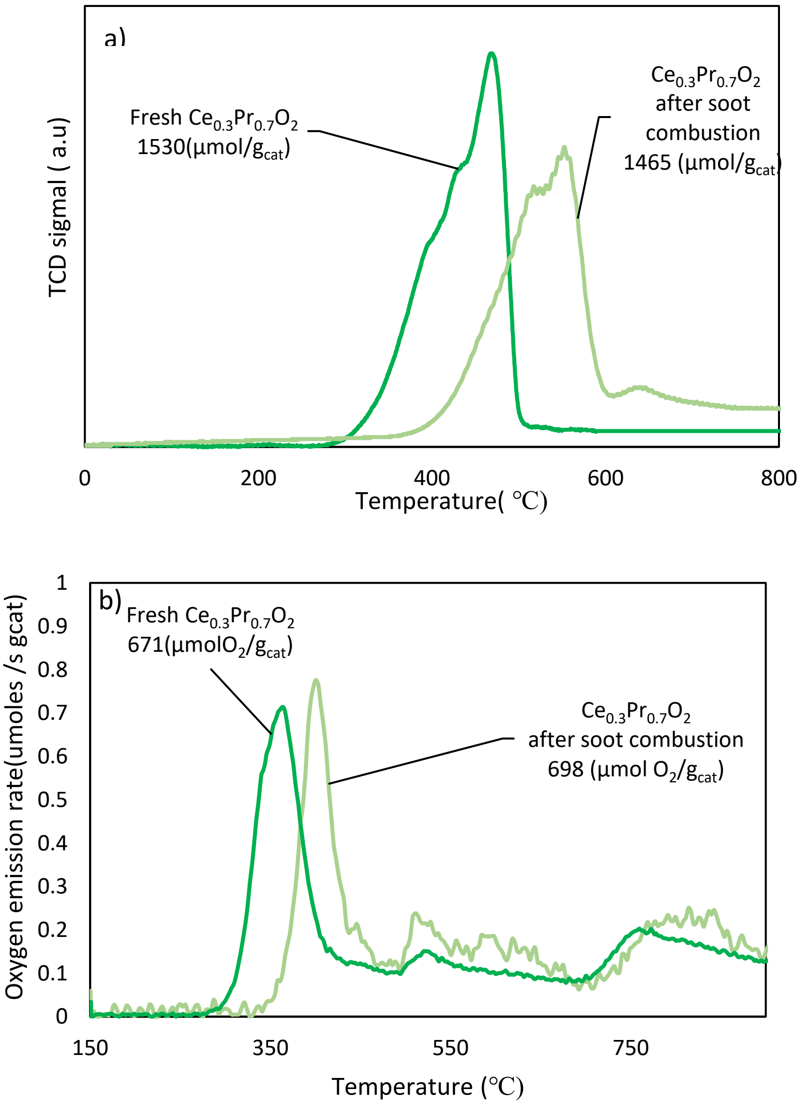

Figure S5. Comparative between fresh and spent catalyst ($Ce_{0.3}Pr_{0.7}O_2$): (**a**) H_2-TPR profiles and (**b**) O_2-TPD profiles.

Upon examination of the oxygen delivery capacity using O_2-TPD under an inert atmosphere (helium), the findings reveal that both the fresh and used $Ce_{0.3}Pr_{0.7}O_2$ catalysts deliver a comparable total amount of O_2. However, slight differences were observed in their respective profiles, with a very minor shift towards higher temperatures noted for the used $Ce_{0.3}Pr_{0.7}O_2$ catalyst (in agreement with the results obtained from H_2-TPR). These results provide further evidence supporting the potential suitability of these formulations for the intended application, highlighting their continued relevance and promising characteristics.

6. References

1. Gamarra, D.; Munuera, G.; Hungrı, A.B.; Ferna, M.; Martı, A. Structure - Activity Relationship in Nanostructured Copper - Ceria-Based Preferential CO Oxidation Catalysts. **2007**, 11026–11038.
2. Borchert, H.; Frolova, Y.V.; Kaichev, V.V.; Prosvirin, I.P.; Alikina, G.M.; Lukashevich, A.I.; Zaikovskii, V.I.; Moroz, E.M.; Trukhan, S.N.; Ivanov, V.P.; et al. Electronic and Chemical Properties of Nanostructured Cerium Dioxide Doped with Praseodymium. *J. Phys. Chem. B* **2005**, *109*, 5728–5738, doi:10.1021/jp045828c.
3. Hardacre, C.; Roe, G.M.; Lambert, R.M. Structure, Composition and Thermal Properties of Cerium Oxide Films on Platinum {111}. *Surf. Sci.* **1995**, *326*, 1–10, doi:10.1016/0039-6028(94)00783-7.
4. Bortamuly, R.; Konwar, G.; Boruah, P.K.; Das, M.R.; Mahanta, D.; Saikia, P. CeO2-PANI-HCl and CeO2-PANI-PTSA Composites: Synthesis, Characterization, and Utilization as Supercapacitor Electrode Materials. *Ionics* **2020**, *26*, 5747–5756, doi:10.1007/s11581-020-03690-7.
5. de Rivas, B.; Guillén-Hurtado, N.; López-Fonseca, R.; Coloma-Pascual, F.; García-García, A.; Gutiérrez-Ortiz, J.I.; Bueno-López, A. Activity, Selectivity and Stability of Praseodymium-Doped CeO2 for Chlorinated VOCs Catalytic Combustion. *Appl. Catal. B Environ.* **2012**, *121–122*, 162–170, doi:10.1016/j.apcatb.2012.03.029.
6. Zhang, H.; Guan, D.; Gu, Y.; Xu, H.; Wang, C.; Shao, Z.; Guo, Y. Tuning Synergy between Nickel and Iron in Ruddlesden–Popper Perovskites through Controllable Crystal Dimensionalities towards Enhanced Oxygen-Evolving Activity and Stability. *Carbon Energy n/a*, e465, doi:10.1002/cey2.465.
7. Rao, C.N.R.; Gopalakrishnan, J. *New Directions in Solid State Chemistry*; Cambridge University Press: Cmbridge, UK, 1997; ISBN 978-0-521-49559-2.
8. Atribak, I. CeO_2 y óxidos mixtos CeO_2-ZrO_2 como catalizadores de la combustión de carbonilla en corrientes gaseosas con NOx y O_2. Universidad de Alicante,San Vicente del Raspeig, Spain,2009.
